# Instruments for the assessment of disaster management among healthcare professionals: a scoping review

**DOI:** 10.3389/fpubh.2025.1540743

**Published:** 2025-04-11

**Authors:** Sara Elshami, Ola Yakti, Mohamed Izham Mohamed Ibrahim, Ahmed Awaisu, Mohamed Sherbash, Banan Mukhalalati

**Affiliations:** ^1^Department of Clinical Pharmacy and Practice, College of Pharmacy, QU Health, Qatar University, Doha, Qatar; ^2^Public Health Department, College of Health Sciences, QU Health, Qatar University, Doha, Qatar

**Keywords:** disaster management, healthcare professionals, assessment instruments, validity, reliability

## Abstract

**Objectives:**

The recent disasters have highlighted the importance of healthcare professionals (HCPs) in aiding communities and maintaining consistent services, prompting a global reconsideration of disaster preparedness approaches. This scoping review aimed to identify and evaluate the psychometric properties of the available instruments that measure disaster preparedness and readiness among HCPs.

**Methods:**

A scoping review was conducted using five concepts: disasters, health personnel, preparedness, management, and questionnaire. Three databases were searched for studies published in English. The identified instruments were summarized according to disaster type, disaster management phase, measurement scope/context, and healthcare discipline. The psychometric properties were evaluated according to content validity, response process, internal structure, relation to other variables, and consequences.

**Results:**

The Emergency Preparedness Information Questionnaire (EPIQ) was the most commonly used instrument, while the Provider Response to Emergency Pandemic (PREP) and the Korean version of the Disaster Preparedness Evaluation Tool (DPET) were the most valid instruments. Most instruments have undergone limited psychometric evaluations, primarily focusing on content and internal structure validations, with response process, relation to other variables, and consequences not frequently reported.

**Conclusion:**

The review highlights the lack of well-developed assessment instruments for disaster preparedness in healthcare disciplines, highlighting the need for future research to develop and thoroughly validate such instruments.

**Systematic review registration:**

https://www.researchregistry.com/browse-the-registry#registryofsystematicreviewsmeta-analyses/registryofsystematicreviewsmeta-analysesdetails/638dbba71e82b30021c02680/.

## 1 Introduction

Disasters have become more frequent and severe, presenting significant challenges to global public health and healthcare systems, and affecting all countries (1, 2). The Swiss Re Institute’s 2023 global summary of catastrophes for 2022 recorded 285 disasters, with an estimated death toll of over 35,000, with over 32,600 cases related to natural catastrophes and 2,500 cases related to man-made disasters (3). The overall economic losses are estimated at USD 284 billion (3).

Successful disaster management (DM), which is planning, organizing, and implementing strategies for anticipating, responding to, and recovering from disasters (4) plays a pivotal role in maintaining the resilience of communities (5). Healthcare professionals (HCPs) are indispensable in mitigating the adverse impacts on communities and providing essential medical services to those affected by the disaster (6, 7). Therefore, to evaluate and manage disasters effectively, HCPs must have a certain degree of preparedness, which may be attained by education and training (8). Additionally, HCPs should possess adequate knowledge, and demonstrate a positive and proactive attitude as well as readiness to respond promptly during such events (9). The knowledge, attitudes, and readiness of HCPs to respond to disasters contribute to their effective performance and achieving successful DM (5).

The considerable emphasis on the field of DM has resulted in the design of several assessment instruments that aim to evaluate a wide range of dimensions, including HCPs’ preparedness competencies (10) and the disaster preparedness of the healthcare system (11). Various assessment instruments have been used to examine the preparedness of HCPs for DM, including their knowledge, skills, attitudes, confidence, and willingness to act effectively during times of crisis (12, 13). Some of the developed instruments examined the preparedness of HCPs for disasters in a general context (10, 14–16), while others targeted their preparedness for particular types of disasters (17–20). However, despite the availability of these tools, there has been limited attention to their psychometric properties.

Psychometric properties, such as reliability and validity, are fundamental indicators of an instrument’s scientific rigor and practical utility (21). Reliability refers to the consistency of an instrument’s results when administered under identical conditions, ensuring that the tool produces stable and reproducible measurements (22). Validity, on the other hand, assesses whether the instrument accurately measures the intended concept, ensuring that it captures all its relevant aspects without distortion (23). A psychometrically sound instrument ensures that preparedness assessments yield meaningful, comparable, and actionable data, which is critical for guiding healthcare policies, disaster response strategies, and targeted training programs (21, 24, 25).

It is worth noting, however, that despite the recognized need for standardized and scientifically sound assessment tools in DM, no prior review has systematically identified and evaluated the psychometric properties, such as reliability, validity, and overall quality, of instruments specifically designed to assess disaster preparedness and readiness of HCPs from different healthcare disciplines. Existing reviews have primarily focused on measuring levels of disaster preparedness among HCPs (26–29) or healthcare systems/agencies (30, 31) or on evaluating the effectiveness of disaster training programs (32). For example, Labrague et al. (26) and Su et al. (27) highlighted significant gaps in HCP preparedness, particularly the impact of prior training and psychological readiness on disaster response effectiveness. Similarly, Said and Chiang (28) identified the need to strengthen both knowledge and competencies among nurses, emphasizing that disaster preparedness extends beyond technical skills to include mental resilience. McCourt et al. (29) extended this analysis to pharmacists and pharmacy students, revealing limited preparedness and a lack of standardized assessment approaches within this professional group.

At the organizational level, Beyramijam et al. (30) identified inadequate preparedness in emergency medical service (EMS) agencies worldwide, highlighting the need for improved preparedness elements such as training, coordination, and policy implementation. Farah et al. (31) highlighted ongoing vulnerabilities in hospital disaster preparedness across sub-Saharan Africa, attributing challenges to governance issues, insufficient funding, and workforce shortages, all of which impede effective disaster response. These findings highlight the broader systemic challenges that impact disaster preparedness beyond individual competency. On the other hand, Williams et al. (32) conducted a systematic review on the effectiveness of disaster training programs, however, the authors noted methodological inconsistencies that limit the ability to draw definitive conclusions regarding their impact on HCPs preparedness. Only one systematic review has specifically examined the quality of instruments designed to assess disaster preparedness of hospitals (33). Heidaranlu et al. (33) noted that existing hospital preparedness tools focus primarily on structural aspects, with little attention to functional capabilities or psychometric validation.

While these studies provide valuable insights into disaster preparedness of HCPs or organizations, and training effectiveness, none have systematically identified and critically assessed the quality of the instruments used in these evaluations. A thorough psychometric evaluation is essential, as existing instruments may yield misleading or inconsistent results if their reliability and validity are not well established. Inaccurate assessments of HCPs’ disaster preparedness could hinder evidence-based improvements in disaster training, policy development, and practical preparedness efforts. Therefore, this scoping review aims to identify and analyze the available instruments that measure disaster preparedness and readiness among HCPs. Additionally, it seeks to evaluate the psychometric properties of these instruments to determine their reliability, validity, and overall quality. This scoping review adopts the Population, Concept, and Context (PCC) framework to define its scope. The population includes HCPs, from any health discipline, including medicine, nursing, pharmacy, paramedicine, public health, and other allied health professions, who play a critical role in DM. The concept focuses on the assessment instruments that assess HCPs’ preparedness and readiness across a broader scope of disasters, with an emphasis on their psychometric properties. The context includes healthcare settings globally, including hospitals, primary healthcare centers, and emergency response units where disaster management is relevant, without restrictions on geographic location or healthcare system type to ensure comprehensive coverage. Unlike previous reviews, this review addresses a critical gap in the comprehensive analysis of the scientific rigor of these instruments. This review enhances the field by offering a structured framework for selecting validated assessment tools, ultimately supporting evidence-based training, policy development, and improved disaster preparedness among HCPs.

## 2 Methods

### 2.1 Protocol registration

Under the registration number [reviewregistry1489], the protocol for this scoping review was filed at the Research Registry (34). The 2018 PRISMA statement for scoping reviews (PRISMA-ScR) is complied with by this scoping review (35).

### 2.2 Eligibility criteria

Only original research articles, theses, and dissertations that reported the assessment of any outcome measures that reflect preparedness and readiness (e.g., competence, knowledge, skills, attitude, or willingness to practice) among HCPs in any health discipline (e.g., medicine, nursing, pharmacy, or other allied health professions, including public health) were included in this review. Instruments originally developed for healthcare professional students were also considered if they were applicable to practicing HCPs. Moreover, articles were included if they reported the utilization of quantitative or mixed-method study designs to develop a new instrument, or adaptation or adoption of existing instruments to assess the preparedness of HCPs for DM. Articles were included if they presented DM instruments with broad applicability rather than those designed for a single, highly specific disaster event. This included tools explicitly designed for disasters in general, without restriction to particular types of disasters, as well as instruments that demonstrated relevance across multiple disaster scenarios. Furthermore, instruments were included if they were designed for specified clustered disaster types, such as biological disasters, provided they were not exclusively developed for a single pathogen or agent. Conversely, articles presenting instruments designed exclusively for particular disasters, such as cholera, smallpox, or bubonic plague, or for specific biological agents, like the coronavirus, were excluded. These instruments were considered too narrowly focused and lacked the broader applicability required for inclusion in the review. Additionally, articles focusing only on routine emergencies, defined as individual-level medical incidents typically occurring in hospital settings that do not constitute public health disasters, were also excluded. Articles were excluded if they reported studies that were conducted in non-health professions or used only qualitative methods. Furthermore, articles were excluded if there was no adequate information about the instrument development and evaluation processes. Moreover, other types of articles that are not original research articles or theses and dissertations were also excluded from this review (e.g., commentaries, pre-print/in-process, editorials).

### 2.3 Information sources

A multidisciplinary team with expertise in public health, disaster management, social and administrative pharmacy practice, health professions education, and scoping review studies developed and revised the search approach. One research team member (SE) performed the search of the literature in October 2022, using PubMed, CINAHL, and ProQuest Public Health. The reference lists of the included articles led to the identification of more articles.

### 2.4 Search strategy

Five key concepts were used: disasters, health personnel, preparedness, management, and questionnaires. The Boolean connector (AND) was used to combine these concepts. Several keywords were utilized to search for each concept. The Boolean connector (OR) was utilized to combine keywords used for each concept. Keywords were applied according to each database, matching them to indexing phrases unique to that database. The year of publication was left unrestricted; however, the search was limited to the English language. Complete search strategies for all databases are shown in Supplementary material 1.

### 2.5 Selection of evidence sources

All identified study citations were imported from the searched databases into the Endnote initially for duplicate identification and then to Covidence© platform for further duplicate identification, article screening and data extraction. After duplicates were removed, an independent assessment of the studies’ eligibility was conducted through titles and abstracts screening of the imported articles by two members of the research team (SE and OY). Eligibility assessment was performed independently by two investigators (SE and OY) on the full text of the included articles. Cases of disagreements in the title and abstract screening and in the full-text screening were resolved through discussions between the two investigators, and consultation with a third investigator (BM). Interrater reliability agreement between the decisions of the investigators was determined for both screening phases through Covidence©.

### 2.6 Data charting process and data items

Covidence© was used to extract, organize and record information obtained from the selected articles. Data extraction comprised: (1) article information (title, author(s), year of publication, and country), (2) study information (study aim and design, number of participants, and the investigated healthcare profession), (3) disaster information [type of disaster, DM phase (general to all phases or specific to one phase)], (4) instrument information [name, type of instrument (originally developed, adopted, or adapted), number of items, assessment outcomes (e.g., knowledge, attitude, willingness, skills, or multiple outcomes), instrument development process, use of theoretical or competency framework, and instrument psychometric measures]. Two investigators (SE and OY) initially piloted the data extraction using sample articles of those included in this review to assess the applicability, identify potential issues, and apply the necessary adjustments before its full-scale implementation. This approach was followed to enhance the reliability and consistency of data extraction when applied to the entire set of reviewed articles. Following successful piloting, the full data extraction was carried out by the two investigators independently.

### 2.7 Critical appraisal of individual sources of evidence

The quality of instruments development and evaluation of their validity and reliability evidence was conducted using the American Psychological and Education Research Associations (APERA) published standards of validity evidence: (1) content, (2) response process, (3) internal structure, (4) relation to other variables, and (5) consequences (36), and using Beckman et al. (37) interpretation of these standard categories. The AERA standards offer a comprehensive, multi-faceted approach to evaluating the validity of instruments (36). It encourages collecting evidence from multiple perspectives to ensure that tools accurately measure their intended aspects, promoting meaningful interpretation and application in diverse contexts (36). Beckman et al. (37) interpretation of these standard categories has been previously applied in various systematic reviews (38, 39). Each standard category was assessed by an assigned rating of N, 0, 1, or 2. The overall rating for each assessment instrument was determined by computing the total sum of asterisks corresponding to the rating of each standard category: N represented zero asterisks, 0 represented one asterisk, 1 represented two asterisks, and 2 represented three asterisks. The definitions of Beckman et al. (37) interpretations of standard categories are listed in Supplementary material 2.

### 2.8 Synthesis of results

Data extracted from the included papers was summarized using descriptive numeric analysis based on the numbers of (1) healthcare discipline, (2) contexts of disasters for which the instrument was used, (3) theoretical or competency framework applied, and (4) psychometric measures. Furthermore, a narrative description of the highly rated and often used instruments was included in the analysis of the extracted data.

## 3 Results

**Figure 1:**
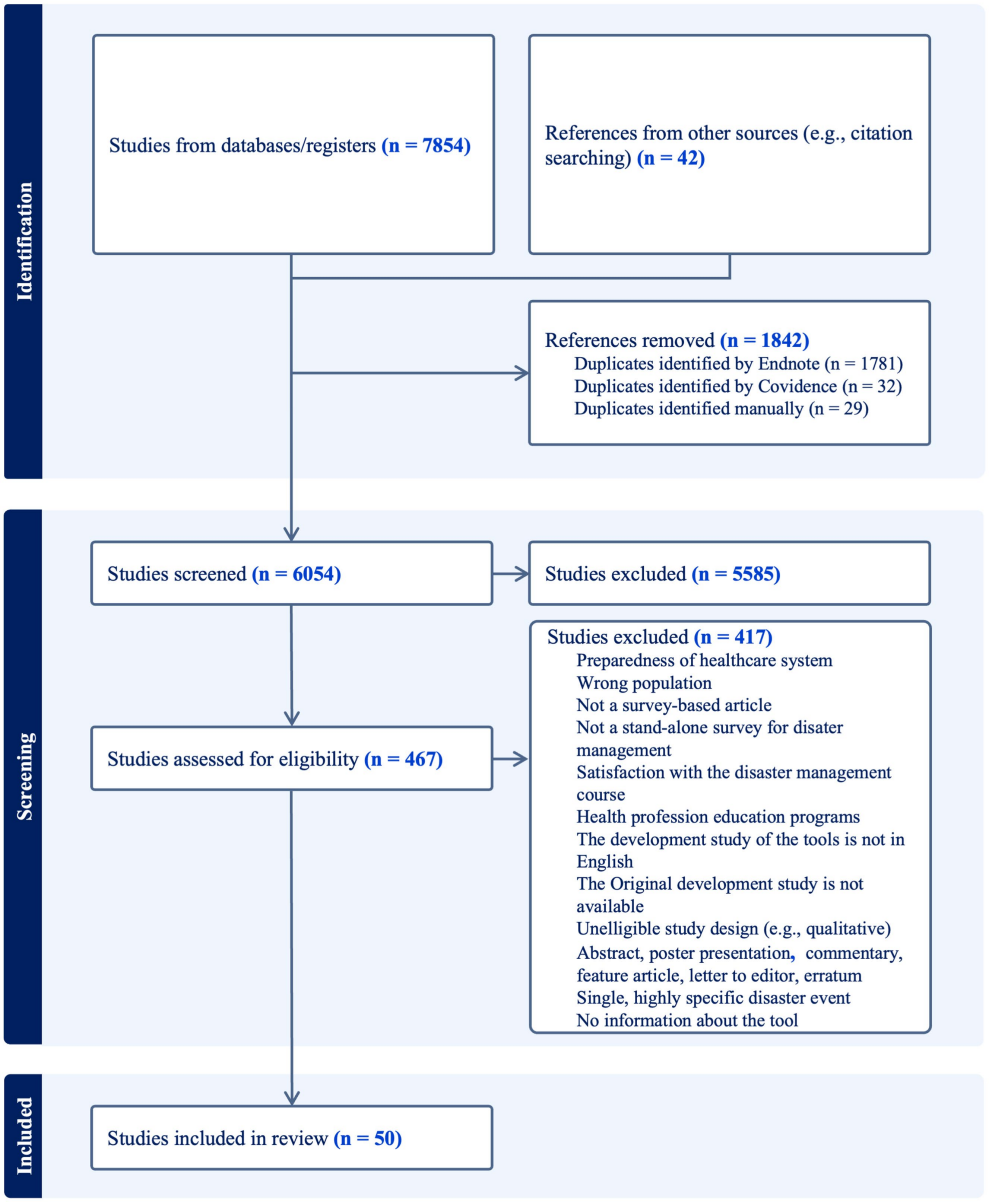
Literature search flow diagram.

Figure 1 illustrates the PRISMA flowchart of the article selection process. The search strategy identified 7,854 articles from databases. An additional 42 articles were retrieved through reference searching. Of the total of 7,896 articles, 1,842 duplicates were identified and subsequently removed. After conducting the title and abstract screening of the remaining 6,054 articles, 467 articles were deemed eligible for full-text screening. The full-text screening resulted in a total of 50 articles that met the inclusion criteria and were utilized in this scoping review. Exclusions included articles outside the scope of primary research literature (e.g., editorial letters, commentaries, protocols), articles that did not describe survey-based research, articles focused on healthcare system preparedness rather than HCPs, studies that assessed DM in populations other than HCPs, and articles that described instruments used to assess the preparedness of HCPs for routine emergency care or to assess the satisfaction of HCPs with DM courses/programs. Many articles were also excluded due to the lack of adequate information about the utilized instrument, or because the originally adopted instruments either did not meet the eligibility criteria of this review or were not published or found online. Moreover, numerous instruments designed for objective assessment of HCPs preparedness for disaster caused by one particular kind of disease were excluded (e.g., assessment of accurate knowledge of COVID-19). The proportionate agreement among investigators for title and abstract screening was 0.80, and for full-text screening it was 0.87, indicating a strong level of proportional agreement.

### 3.1 Characteristics of the included studies

Out of the 50 included articles, 32 described the development of a new instrument (i.e., originally developed) (2, 9, 10, 12, 13, 15, 16, 20, 40–63), while three articles reported the adaptation (64–66), and 15 articles reported the adoption of existing instruments for assessing the preparedness of HCPs during disasters (7, 67–80). The 32 articles describing a new instrument development were published between 2002 and 2022. Notably, the highest proportion of articles were published after 2015, with 2019 (12.5%) and 2020 (9.4%) being particularly notable years. Of these, 13 described studies conducted in the USA (2, 12, 13, 20, 40, 43, 49, 52, 53, 57, 59–61), eight described studies conducted in Middle Eastern countries (i.e., Saudi Arabia, Yemen, Qatar, Jordan, and Iran) (9, 10, 15, 41, 45–47, 54), and two described studies conducted in Nigeria (48, 62). One described a study conducted in Canada (58), China (51), India (44), Ireland (16), Taiwan (50), Philippines (55), Ethiopia (56), Brazil (42), and Europe-wide countries (63). Of the 32 articles reporting new instrument development, only two described studies that utilized a mixed-methods study design (20, 57). Furthermore, 11 studies assessed preparedness and readiness to practice during disasters among multi-professions (9, 15, 16, 20, 41, 45, 47, 49, 53, 56, 58), 11 among nursing profession (2, 10, 13, 40, 42, 43, 50, 51, 54, 57, 59), two among dentistry profession (44, 61), two among pharmacy profession (62, 63), and one each in medicine profession (60), emergency medical services profession (46), occupational therapy profession (55), physiotherapy profession (48), anesthesiology profession (52), and environmental health profession (12). On the other hand, the three articles that reported the use of adapted instruments were published in 2008 (64) and 2010 (65, 66), and the studies were conducted in the USA (64), Jordan (65), and Korea (66), among nurses. The articles that reported the adoption of pre-existing instruments (*n* = 15) were published between 2012 and 2022, with the highest proportion of publications occurring in 2020 (26.7%), followed by 2015 (13.3%) and 2012 (13.3%). These studies were conducted across various countries, including Indonesia, Pakistan, Nigeria, and Iran. These instruments were not discussed in depth because they did not contribute additional information concerning the development or evaluation of the instruments.

### 3.2 Characteristics of the included instruments

Examples of the identified instruments included in this review are the Emergency Preparedness Information Questionnaire (EPIQ) (40), Disaster Preparedness Evaluation Tool (DPET) (2), Knowledge, Attitude, Readiness to Practice (KArP) (15), Disaster Nursing Core Competencies Scale (DNCCS) (10), Major Emergency Preparedness in Ireland Survey (MEPie) (16), Provider Response to Emergency Pandemic (PREP) (20), Nurses’ Disaster Response Competencies Assessment Questionnaire (NDRCAQ) (42), and Domestic Preparedness Questionnaire (DPQ) (49). The use of a theoretical or competency framework was reported in the development of 12 instruments (2, 10, 12, 13, 16, 42, 43, 47, 50, 52, 56, 63). The most commonly used competency framework to develop the assessment instruments in the included articles is the ‘Framework of Disaster Nursing Competencies’, which was collaboratively developed by the International Council of Nurses (ICN) and the World Health Organization (WHO) (81). Fifteen of the instruments described in the included studies assessed preparedness regarding disasters in general (9, 10, 12, 15, 16, 40–42, 44, 48, 50, 51, 55, 62, 63), while 11 instruments focused on preparedness for a specific type of disasters (e.g., a disaster from a biological source) (2, 9, 13, 20, 43, 46, 53, 54, 59–61), and six instruments focused on preparedness for multiple specified disasters (e.g., chemical, biological, radiological, nuclear, and explosive) (47, 49, 52, 56–58). The majority of the instruments in the included studies assessed the disaster preparedness of HCPs across multiple DM phases, and only four instruments focused on determining the disaster preparedness and readiness of HCPs within a specific DM phase (e.g., response) (42, 43, 47, 49). Most assessment instruments in the included studies examined competencies for DM by evaluating the knowledge, skills, attitudes, confidence, and readiness/willingness/concerns to practice HCPs. The included studies that reported the adaptation of pre-existing instruments were used for the same scope as the originally developed instruments. The included studies that reported the adoption of pre-existing instruments for assessing the preparedness of HCPs during disasters were distributed as follows: six articles adopted EPIQ (67–71, 75), two adopted the original DPET (72, 73) and one adopted AlKhalaileh et al. (65) version of DPET, four adopted Rajesh et al. instrument (76–79), one adopted the DNCCS (7), and one adopted the KArP instrument (74). Supplementary material 3 represents a summary of the included articles. The articles that reported the adoption of pre-existing instruments (*n* = 15) were not reported in the table since they do not contribute additional information concerning the development or evaluation of the instruments.

### 3.3 Validity and reliability evidence of the assessment instruments

The findings suggested that most instruments have limited validity and reliability evidence, including reporting only content validity and/or internal consistency reliability out of the five categories. For the ‘content’ category, most articles described the patterns, language, and structure of items, as evaluated by experts and the target population. Of note, content evaluation was conducted subjectively in most articles by a narrative qualitative evaluation of the content. In contrast, only a few articles reported objective measures of content evaluation, such as calculating the content validity index. For the ‘internal structure’, most of the articles reported at least one measure of reliability (e.g., internal consistency), and some articles reported using factor analysis (e.g., exploratory factor analysis).

The categories ‘response process’ and ‘relation to other variables’, which report critical examinations of thought processes and response error for ‘response process’ and convergence or divergence between assessment scores for ‘relation to other variables’, were the least reported standard categories. For the ‘response process’ criterion, almost all articles reported the response rate for the main study employing the assessment instrument and not the response rate for pilot testing of the instrument. In addition, no or minimal discussion on the thought processes, analysis of responses, or response errors were presented. The convergent or discriminating correlations between disaster preparedness scores and other factors pertinent to the assessed construct were only reported in a small number of papers under the ‘relation to other variables’ category. Furthermore, none of the instruments in the included articles assessed the ‘consequence’ category which involves evaluation of the effects of the assessment and their impacts on validity.

According to APERA published standards of validity evidence and Beckman et al. (37) interpretation used in this review, the higher the number of asterisks given to an assessment instrument, the more valid and reliable the instrument is. The most valid and reliable instruments in this review were PREP and the Korean version of DPET. Out of the 32 articles that described the development of a new instrument, eight of those instruments attained an overall rating of one asterisk, six attained two asterisks, two attained three asterisks, and the rest attained four or more asterisks. Supplementary material 3 presents a summary of the psychometric properties of the instruments in the included articles. Further details about the evaluation evidence of the psychometric properties are presented in Supplementary material 4.

The following description provides an overview of PREP and the other frequently used assessment instruments.

#### 3.3.1 Provider response to emergency pandemic (PREP)

PREP was the most valid and reliable assessment instrument in the included articles. Good (20) developed PREP to determine the willingness of HCPs in the USA to continue working in case of a biological disaster. The instrument development was based on four loss subscales (loss of order, safety, trust, and freedom) and five items assessing the sense of duty and loyalty, resulting in 31 items. Responses were collected using a four-point Likert scale of agreement. The subscales had good internal consistency reliability.

#### 3.3.2 Emergency preparedness information questionnaire (EPIQ)

EPIQ is a widely used instrument developed by Wisniewski et al. (40) in the USA to assess nurses’ disaster preparedness skills, employing a five-point Likert scale of familiarity related to 44 items. EPIQ was originally developed as eight distinct dimensions: identification, incident command system, triage, epidemiology and monitoring, isolation, decontamination and quarantine, communication, psychological considerations, and reporting. The internal consistency reliability of the questionnaire was considered good. In 2008, the psychometric properties of EPIQ were re-evaluated by Garbutt, Peltier, and Fitzpatrick, who performed a principal component analysis (PCA), and a revised version of EPIQ was established (64).

#### 3.3.3 Disaster preparedness evaluation tool (DPET)

DEPT, a commonly used instrument, was developed in 2009 by Bond and Tichy in the USA to evaluate the knowledge and competencies of nurse practitioners in disaster preparedness, as well as their response and management capabilities (2). The instrument was designed based on the disaster preparedness competencies specified in the 1996 edition of the American Association of Colleges of Nursing’s Essentials of Master’s Education. The level of disaster preparedness was assessed through 47 items, utilizing a Likert scale of agreement. The internal consistency analysis demonstrated excellent reliability across various domains. In Jordan and Korea, PCA was conducted for further psychometric evaluation of the instrument. In Jordan, the analysis of PCA resulted in three factors: knowledge, skills and post-DM, with an excellent internal consistency reliability (65). Whereas, in Korea, the PCA resulted in five factors: disaster education and training, disaster knowledge and information, bioterrorism and emergency response, disaster response, and disaster evaluation, with acceptable internal consistency reliability analysis (66).

#### 3.3.4 Disaster nursing core competencies scale (DNCCS)

DNCCS, one of the most commonly used instruments, was developed by Al Thobaity et al. (10) to explore the fundamental skills in disaster nursing, the roles undertaken by nurses in DM, and the obstacles to advancing disaster nursing in Saudi Arabia. The International Council of Nurses (ICN) disaster-nursing framework was the guide for the development of DNCCS. Through the PCA, DNCCS distinctly represented three key core factors: essential competencies, the obstacles encountered by disaster nurses in the KSA, and the role of nurses in DM. Overall, the scale had excellent internal consistency reliability.

#### 3.3.5 Knowledge, attitude, and readiness to practice (KArP)

In 2020, Al-Ziftawi et al. developed the KArP instrument, which is also one of the most commonly used instruments, to evaluate the level of knowledge, attitude, and readiness to practice disaster medicine and preparedness among health professional students in Qatar (15). The questionnaire comprised three key domains: knowledge (22 yes/no questions), attitude (16 questions on a 5 Likert scale of agreement), and readiness to practice (11 questions on a 5 Likert scale of agreement), in addition to a not applicable option. The KArP instrument exhibited an overall excellent internal consistency reliability.

## 4 Discussion

This review offers a thorough examination of instruments currently in use for assessing DM among HCPs. It enhances comprehension regarding the evaluation of HCPs’ preparedness and readiness for disasters, while also raising concerns about the quality of the assessment instruments employed.

Analyzing the geographic spread of studies revealed important information into how HCPs disaster preparedness and readiness assessment instruments have been developed, adapted, and adopted across diverse healthcare environments. The findings indicated that new instrument development primarily occurred in high-income countries, particularly the United States, where structured disaster management programs and extensive healthcare resources may influence instrument design and validation. In contrast, Middle Eastern and African countries have made contributions to instrument development which shows their growing awareness of disaster preparedness within their emerging healthcare systems. Furthermore, a review of publication trends highlighted a growing emphasis on both the development of new DM assessment instruments and the adoption of existing instruments. Recent global crises and the pressing need to enhance disaster resilience have likely resulted in increased attention. The rising number and complex nature of disasters have made validated assessment measures necessary to establish strong evaluation frameworks which will help HCPs become better prepared and, ultimately, the resilience of healthcare systems.

This review suggests that most assessment instruments focused on assessing the preparedness and readiness of HCPs for disasters in general, with less emphasis on specific disaster types like disasters from biological sources. This aligns with a systematic review by Said and Chiang (28) that examined nurses’ knowledge, skill capabilities, and psychological preparedness for disasters. The review revealed areas of inadequate knowledge perceived among nurses, particularly biological information and bioterrorism handling, which might necessitate frequent evaluation and monitoring (28). It is worth noting that many of the instruments discussed in the included articles in this review were profession-specific, with limited applicability to other healthcare professions. In that regard, Daily et al. (82) review of the literature concerning disaster competencies among HCPs revealed numerous inconsistent competencies with imprecise terminology and structure, and the absence of universal consensus for any of these competencies. Challenges in developing competencies that apply to broader healthcare professions include diverse roles and varying degrees of proficiency in certain competencies (82).

The review reveals that many studies on assessing DM among HCPs have focused on a limited set of validity measures and psychometric evaluation (9, 12, 52–63). Those instruments predominantly emphasized ‘content’ and ‘internal structure’ validities, whereas ‘response process,’ ‘relation to other variables,’ and ‘consequences’ validities received lower attention. This lack of comprehensive evidence often hinders researchers from selecting the most suitable assessment instrument, raising concerns about the credibility of conclusions drawn from existing assessments. Those concerns are consistent with those raised by Beckman et al.’s (37) review of assessment instruments used for clinical teaching (37).

This scoping review suggested that content validity was the most reported validity measured in the included articles. Most articles adopted a process to ensure that the structure, language, and ideas of the items accurately assess the construct of interest. The development of items in the majority of instruments was not theoretical or competency framework-driven, which is consistent with Heidaranlu et al.’s (33) review. In that regard, the American Psychological Association has reinforced the significance of grounding construct development within a theoretical framework to ensure the validity of the assessment (36). The results of this scoping review suggested that the ‘internal structure’ was frequently demonstrated in the instruments in included studies, with the majority of studies indicating at least one type of reliability. A similar outcome was also found in a systematic review conducted to evaluate the psychometric measures of the instruments utilized for hospital disaster preparedness using COSMIN criteria (33), which demonstrated that the retrieved instruments focused on assessing reliability (33). This finding can be explained by the ability to conduct reliability testing on preexisting data without prior planning (37). On the other hand, studies must be purposefully designed to evaluate hypothesized associations to enable the analysis of the ‘relation to other variables’. The absence of pre-analysis planning for these types of assessments may explain the relatively minimal coverage of this category in the included studies (37). Examples of meaningful associations between the preparedness assessment and readiness assessment of HCPs and other variables may involve positive or negative relationships between preparedness scores and outcomes such as DM and may also illustrate potential relationships between scores from two distinct assessment instruments (37).

This review revealed that nearly all articles focused on the response rate for the main study using the assessment instrument, without discussing the rationale behind these responses or potential response errors. Response errors are typically related to the sample size, with larger samples resulting in lower errors (83, 84). For example, in this review, Al Khalaileh et al. (65) implemented the recommended sample size of five to ten respondents per item as recommended by Nunnally and Bernstein (85), with Field (86) claiming that a sample of more than 300 is adequate to ensure the reliability of factor analysis. Nevertheless, none of the studies has critically examined the implication of sample size on response errors or analyzed the responses for evidence of halo error, or rater leniency, or data demonstrating low response error. Additionally, none of the instruments in the included articles evaluated the intended or unintended effects of DM assessment. Evaluating the outcome of assessments can reinforce the credibility of the scores (37). According to Eignor, evidence for ‘consequence’ validity should explicitly demonstrate the consequences of an assessment and the impact of these consequences on the interpretation of scores.

### 4.1 Limitations

The review uses APERA-published standards of validity evidence, a reliable method for evaluating assessment instrument validity. However, the review has limitations, including the inability to use the COSMIN checklist due to its cognitively demanding nature and the use of only three databases, potentially omitting important articles. However, to lessen the impact of this potential exclusion, an assessment of the included articles’ reference lists was done to find pertinent articles. Moreover, this review focused on disaster assessment tools with broad applicability and excluded instruments developed exclusively for particular disasters, such as cholera, or smallpox, or for specific biological agents, like the coronavirus. While this approach ensures a comprehensive evaluation of widely applicable assessment tools, it may limit insights into the effectiveness of disaster-specific instruments that could offer valuable context-specific assessments.

### 4.2 Future recommendations and implication for policy and practice

This review calls for further research that fosters the development of more robust assessment instruments that are theory/competency-based, are adequately evaluated for evidence of broader validity (e.g., relation to other variables and consequences), and hold significance to various health disciplines, in order to meet the evolving challenges of DM. HCPs could potentially benefit from formulating universally applicable DM competencies that are relevant to various healthcare fields. It is also important that researchers recognize the significance of adopting assessment instruments that are grounded in substantial evidence of validity and reliability, and not only those that are most frequently used. Relying on valid and reliable assessment instruments will ultimately assist stakeholders in highlighting key areas for improvement and innovation, and optimizing training programs, resource allocation, and strategic planning for disaster situations. Future research could also explore the applicability and adaptability of disaster assessment instruments developed for specific disaster scenarios, such as COVID-19, to determine whether these tools can be effectively utilized in other public health emergencies. Additionally, further studies may investigate how such instruments perform beyond their originally intended disaster context.

## 5 Conclusion

This review provided an in-depth summary of the assessment instruments used to evaluate the readiness and preparedness of HCPs during disasters in different healthcare sectors, and it illustrated a brief overview of the psychometric properties of those instruments. The assessment tools identified for evaluating DM among HCPs exhibited notable strengths across various dimensions. For example, EPIQ has been extensively employed to assess general disaster preparedness which provides a robust framework for evaluating DM capabilities. Similarly, MEPie has explored perceptions of emergency planning among diverse emergency responders which offers insights from various categories of HCPs. Additionally, advanced validation techniques, such as confirmatory factor analysis, were applied to the adapted version of DPET, enhancing the instrument’s validity in assessing disaster preparedness. Moreover, tools like DNCCS, developed and evaluated in Arab countries, present valuable opportunities to examine and address DM assessment within an Arabic context. Despite these strengths, this review indicated that most instruments in the included articles have undergone minor psychometric evaluations, predominantly emphasizing the ‘content’ and ‘internal structure’ validities, whereas ‘response process,’ ‘relation to other variables’ and ‘consequences’ validities received the lowest attention. The review identified a critical absence of tools demonstrably developed and rigorously assessed for broad implementation across various healthcare specialties and disaster scenarios.

## Data Availability

The original contributions presented in the study are included in the article/Supplementary material, further inquiries can be directed to the corresponding author.
