# Supplementary material for: Instruments for the assessment of disaster management among healthcare professionals: a scoping review
Source: Front Public Health. 2025 Apr 11;13:1540743. doi: 10.3389/fpubh.2025.1540743 (PMC12021930; doi:10.3389/fpubh.2025.1540743)
Supplement: Supplementary file 1 [file Table_1.docx]

**Supplementary Material 1. Search strategies for all searched databases**

| **Database** | **Search** | **Date** | **Filters** | **Hits** |
| --- | --- | --- | --- | --- |
| **Pubmed** | (("disasters"[MeSH Terms] OR "disaster medicine"[MeSH Terms] OR "cris*"[Title/Abstract] OR "emergen*"[Title/Abstract]) AND "english"[Language] AND ("health personnel"[MeSH Terms] AND "english"[Language]) AND (("competen*"[Title/Abstract] OR "knowledg*"[Title/Abstract] OR "aware*"[Title/Abstract] OR "attitude of health personnel"[MeSH Terms] OR "skill*"[Title/Abstract] OR "readiness*"[Title/Abstract] OR "prepar*"[Title/Abstract] OR "willing*"[Title/Abstract] OR "confiden*"[Title/Abstract]) AND "english"[Language]) AND (("tool*"[Title/Abstract] OR "instrument*"[Title/Abstract] OR "survey*"[Title/Abstract] OR "quantitative*"[Title/Abstract] OR "questionnaire*"[Title/Abstract] OR "scale*"[Title/Abstract] OR "inventor*"[Title/Abstract]) AND "english"[Language]) AND (("plan*"[Title/Abstract] OR "predict*"[Title/Abstract] OR "prevent*"[Title/Abstract] OR "recover*"[Title/Abstract] OR "respon*"[Title/Abstract] OR "manag*"[Title/Abstract] OR "control*"[Title/Abstract] OR "resilien*"[Title/Abstract] OR "protect*"[Title/Abstract] OR "reduc*"[Title/Abstract]) AND "english"[Language])) AND (english[Filter]) | 4 October 2022 | English | 4116 |
| **CINAHL** | (TI(disaster*) OR AB(disaster*) OR TI(cris*) OR AB(cris*) OR TI(emergenc*) AB(emergenc*) OR TI(“disaster medicine”) OR AB(“disaster medicine”)) AND (TI(“Healthcare provider*”) OR TI(“health care provider*”) OR TI(“Health personnel” ) OR TI(“health care personnel”) OR TI(“healthcare personnel”) OR TI(“healthcare practitioner*”) OR TI(“health care practitioner*”) OR TI("Health profession*") OR TI("Healthcare profession*") OR TI("Health care profession*") OR TI("Allied health") OR TI(“hospital staff”) OR TI(“healthcare staff”) OR TI(“health care staff”) OR TI("Health science*") OR TI(Biomed*) OR TI(dent*) OR TI(nurs*) OR TI(nutrition*) OR TI(diet*) OR TI(physiotherap*) OR TI(“occupation therap*”) OR TI(radiotherap*) OR TI(“radiation therap*”) OR TI(“physical therap*”) OR TI(medic*) OR TI(doctor*) OR TI(clinician*) OR TI(surg*) OR TI(pharmac*) OR TI(audiolog*) OR TI(psych*) OR TI(midwi*) OR TI(physician*) OR TI(“public health”) OR AB(“Healthcare provider*”) OR AB(“health care provider*”) OR AB(“Health personnel”) OR AB(“health care personnel”) OR AB(“healthcare personnel”) OR AB(“healthcare practitioner*”) OR AB(“health care practitioner*”) OR AB("Health profession*") OR AB("Healthcare profession*") OR AB("Health care profession*") OR AB("Allied health") OR AB(“hospital staff”) OR AB(“healthcare staff”) OR AB(“health care staff”) OR AB("Health science*") OR AB(Biomed*) OR AB(dent*) OR AB(nurs*) OR AB(nutrition*) OR AB(diet*) OR AB(physiotherap*) OR AB(“occupation therap*”) OR AB(radiotherap*) OR AB(“radiation therap*”) OR AB(“physical therap*”) OR AB(medic*) OR AB(doctor*) OR AB(clinician*) OR AB(surg*) OR AB(pharmac*) OR AB(audiolog*) OR AB(psych*) OR AB(midwi*) OR AB(physician*) OR AB(“public health”)) AND (TI(Competen*) OR TI(knowledg*) OR TI(aware*) OR TI(attitud*) OR TI(skill*) OR TI(readiness*) OR TI(prepar*) OR TI(willing*) OR TI(confiden*) OR AB(Competen*) OR AB(knowledge*) OR AB(aware*) OR AB(attitud*) OR AB(skill*) OR AB(readiness*) OR AB(prepar*) OR AB(willing*) OR AB(confiden*)) AND (TI(tool*) OR TI(instrument*) OR TI(survey*) OR TI(quantitativ*) OR TI(questionnaire*) OR TI(scale*) OR TI(inventor*) OR AB(tool*) OR AB(instrument*) OR AB(survey*) OR AB(quantitativ*) OR AB(questionnaire*) OR AB(scale*) OR AB(inventor*)) AND (TI(plan*) OR TI(predict*) OR TI(prevent*) OR TI(recover*) OR TI(respon*) OR TI(manag*) OR TI(control) OR TI(resilien*) OR TI(protect*) OR TI(reduce*) OR AB(plan*) OR AB(predict*) OR AB(prevent*) OR AB(recover*) OR AB(respon*) OR AB(manag*) OR AB(control) OR AB(resilien*) OR AB(protect*) OR AB(reduce*)) | 4 October 2022 | English | 2094 |
| **ProQuest Public Health** | (TI(disaster*) OR AB(disaster*) OR TI(cris*) OR AB(cris*) OR TI(emergenc*) AB(emergenc*) OR TI(“disaster medicine”) OR AB(“disaster medicine”)) AND (TI(“Healthcare provider*”) OR TI(“health care provider*”) OR TI(“Health personnel” ) OR TI(“health care personnel”) OR TI(“healthcare personnel”) OR TI(“healthcare practitioner*”) OR TI(“health care practitioner*”) OR TI("Health profession*") OR TI("Healthcare profession*") OR TI("Health care profession*") OR TI("Allied health") OR TI(“hospital staff”) OR TI(“healthcare staff”) OR TI(“health care staff”) OR TI("Health science*") OR TI(Biomed*) OR TI(dent*) OR TI(nurs*) OR TI(nutrition*) OR TI(diet*) OR TI(physiotherap*) OR TI(“occupation therap*”) OR TI(radiotherap*) OR TI(“radiation therap*”) OR TI(“physical therap*”) OR TI(medic*) OR TI(doctor*) OR TI(clinician*) OR TI(surg*) OR TI(pharmac*) OR TI(audiolog*) OR TI(psych*) OR TI(midwi*) OR TI(physician*) OR TI(“public health”) OR AB(“Healthcare provider*”) OR AB(“health care provider*”) OR AB(“Health personnel”) OR AB(“health care personnel”) OR AB(“healthcare personnel”) OR AB(“healthcare practitioner*”) OR AB(“health care practitioner*”) OR AB("Health profession*") OR AB("Healthcare profession*") OR AB("Health care profession*") OR AB("Allied health") OR AB(“hospital staff”) OR AB(“healthcare staff”) OR AB(“health care staff”) OR AB("Health science*") OR AB(Biomed*) OR AB(dent*) OR AB(nurs*) OR AB(nutrition*) OR AB(diet*) OR AB(physiotherap*) OR AB(“occupation therap*”) OR AB(radiotherap*) OR AB(“radiation therap*”) OR AB(“physical therap*”) OR AB(medic*) OR AB(doctor*) OR AB(clinician*) OR AB(surg*) OR AB(pharmac*) OR AB(audiolog*) OR AB(psych*) OR AB(midwi*) OR AB(physician*) OR AB(“public health”)) AND (TI(Competen*) OR TI(knowledg*) OR TI(aware*) OR TI(attitud*) OR TI(skill*) OR TI(readiness*) OR TI(prepar*) OR TI(willing*) OR TI(confiden*) OR AB(Competen*) OR AB(knowledge*) OR AB(aware*) OR AB(attitud*) OR AB(skill*) OR AB(readiness*) OR AB(prepar*) OR AB(willing*) OR AB(confiden*)) AND (TI(tool*) OR TI(instrument*) OR TI(survey*) OR TI(quantitativ*) OR TI(questionnaire*) OR TI(scale*) OR TI(inventor*) OR AB(tool*) OR AB(instrument*) OR AB(survey*) OR AB(quantitativ*) OR AB(questionnaire*) OR AB(scale*) OR AB(inventor*)) AND (TI(plan*) OR TI(predict*) OR TI(prevent*) OR TI(recover*) OR TI(respon*) OR TI(manag*) OR TI(control) OR TI(resilien*) OR TI(protect*) OR TI(reduce*) OR AB(plan*) OR AB(predict*) OR AB(prevent*) OR AB(recover*) OR AB(respon*) OR AB(manag*) OR AB(control) OR AB(resilien*) OR AB(protect*) OR AB(reduce*)) | 4 October 2022 | English | 1644 |
