# Supplementary material for: Instruments for the assessment of disaster management among healthcare professionals: a scoping review
Source: Front Public Health. 2025 Apr 11;13:1540743. doi: 10.3389/fpubh.2025.1540743 (PMC12021930; doi:10.3389/fpubh.2025.1540743)
Supplement: Supplementary file 2 [file Table_2.docx]

### Supplementary Material 2. Beckman et al. (2005) interpretation of APERA standards of validity evidence used to assess the quality of the included instrument

| Test category | Rating | Criteria |
| --- | --- | --- |
| Content | N | No discussion of instrument content (includes simply listing items without justification) |
|  | 0 | Discussion but no data |
|  | 1 | Listing assessment themes with little or no reference to a theoretical basis, or a poorly defined process for creating and reviewing items |
|  | 2 | A well-defined process for developing instrument content, including both an explicit theoretical/conceptual basis for instrument items and systematic item review by experts. Alternatively, reference to a prior study on an assessment instrument that meets these criteria |
| Response process | N | No discussion. Merely disclosing response rates or numbers of respondents does not constitute evidence |
|  | 0 | Discussion but no data. Discussing the impact of response rate on assessment scores, or speculating on the thought processes of learners, does not constitute evidence |
|  | 1 | Minimal data regarding thought processes and analysis of responses. Description (without data) of systems that reduce response error, such as computer-scored forms |
|  | 2 | Multiple sources of supportive data, including critical examination of thought processes, analysis of responses for evidence of halo error or rater leniency, or data demonstrating low response error |
| Internal structure | N | No discussion |
|  | 0 | Discussion but no data |
|  | 1 | Factor analysis incompletely confirming anticipated data structure, or acceptable reliability with a single measure |
|  | 2 | Factor analysis confirming anticipated data structure, or multiple measures of reliability. Variation in responses to specific items among subgroups (differential item functioning) can support or challenge internal structure depending on predictions |
| Relation to other variables | N | No discussion |
|  | 0 | Discussion but no data |
|  | 1 | Correlation of assessment scores to outcomes with minimal theoretical importance, or unanticipated score correlations |
|  | 2 | Correlation (convergence) or no correlation (divergence) between assessment scores and theoretically predicted outcomes or measures of the same construct. Such evidence will usually be integral to the study design, and anticipated a priori |
| Consequences | N | No discussion. Speculation on potential applications of the assessment does not constitute evidence |
|  | 0 | Discussion, but no data. Simply discussing the consequences of assessment (e.g., data regarding usefulness or faculty approval) without linking this to validity does not constitute evidence |
|  | 1 | Description of consequences of assessment that could conceivably impact the validity of score interpretations (although these impacts are not explicitly identified by the authors) |
|  | 2 | Description of consequences of assessment that clearly impact on the validity of score interpretations, as supported by data and convincingly argued by the authors. Such evidence will usually be integral to the study design, and anticipated a priori |
