# Supplementary material for: Instruments for the assessment of disaster management among healthcare professionals: a scoping review
Source: Front Public Health. 2025 Apr 11;13:1540743. doi: 10.3389/fpubh.2025.1540743 (PMC12021930; doi:10.3389/fpubh.2025.1540743)
Supplement: Supplementary file 3 [file Table_3.docx]

### **Supplementary Material 3. Summary of included studies: study characteristics, tool details, and psychometric evaluation**

| **Study details** | | | | **Instrument details** | | | | | | **Validity testing** | | | | | |
| --- | --- | --- | --- | --- | --- | --- | --- | --- | --- | --- | --- | --- | --- | --- | --- |
| **Author (Date)**  **Design** | **Country** | **Profession** | **No. of participants** | **Type of instrument** | **Type of disaster studied** | **General or specific disaster management phase** | **Name of the instrument** | **Instrument sections/ Outcome measure**  **(No. of items)** | **Use of theory / models / competency** | **Content** | **Response process** | **Internal structure** | **Relation to other variables** | **Consequences** | **Total score** |
| Wisniewski R, et. al. (41) (2004)  Quantitative research | USA | Nursing | 877 | Originally developed | Disasters in general | Multiple (e.g., prevention, mitigation, response, recovery) | Emergency Preparedness Information Questionnaire (EPIQ) | - Knowledge of the emergency preparedness competencies (44 items) | No | 1 | N | 2 | N | N | ***** |
| ***Adapted by Garbutt S. et. al. (65) (2008)*** | USA | Nursing | 776 | Adapted | Based on the findings of a principle-components analysis using a varimax rotation, the original EPIQ was revised:   - one question with a factor loading of < 0.4 was removed from consideration - items were moved from the original dimension (factor) to the dimension where the items best fit, based on the factor analysis - resulted in a different set and configuration of dimensions, compared with those reported by Wisniewski et al. | | | | | N | N | 2 | 2 | N | ****** |
| Tichy et al (2) (2009)  Quantitative research | USA | Nursing | 166 | Originally developed | Specific disaster type (disaster of a biological source) | Multiple (e.g., mitigation, preparedness, response, recovery) | Disaster Preparedness Evaluation Tool (DPET) | - Demographics (21 items) - Level of preparedness (25 items; disaster knowledge, disaster skills, and personal preparedness) - Level of preparedness for response (16 items; knowledge and patient management) - Level of preparedness for disaster recovery (6 items; knowledge and management) | Disaster preparedness competencies for nurse practitioners | 1 | N | 1 | N | N | **** |
| ***Adapted by Al Khalaileh M, et. al. (66) (2010)*** | Jordan | Nursing | 474 | Adapted | - Three items were omitted - Beaton et al. (2000) Guidelines for the Process of Cross-Cultural Adaptation of Self-Report Measures were used - Three factor-tool: knowledge, skills and post disaster management | | | | | 2 | 1 | 2 | 1 | N | ********** |
| ***Adapted by Suk Jung Han and Jiyoung Chun (67) (2010)*** | Korea | Nursing | 497 | Adapted | - The DPET-K had 28 items with five factors (disaster education and training, disaster knowledge and information, bioterrorism and emergency response, disaster response, and disaster evaluation) | | | | | 2 | 1 | 2 | 2 | N | *********** |
| Al Thobaity A, et. al. (10) (2016)  Quantitative research | KSA | Nursing | 132 | Originally developed | Disasters in general | Multiple (e.g., mitigation, planning, preparedness and response) | The Disaster Nursing Core Competencies Scale (DNCCS) | - Demographics - Core competencies of disaster nursing (29 items) - Barriers to developing disaster nursing (10 items) - Nurses’ roles in disaster management (5 items) | The International Council of Nurses (ICN) disaster-nursing framework | 2 | N | 2 | N | N | ****** |
| Al-Ziftawi N, et. al. (15) (2020)  Quantitative research | Qatar | Multi-professions | 100 | Originally developed | Disasters in general | Multiple (e.g., preparedness and response) | Knowledge, Attitude, readiness to Practice (KArP) | - Knowledge (22 items) - Attitude (16 items) - Readiness to practice in disaster (11 items) | No | 1 | N | 1 | N | N | **** |
| Veenema T, et al (16) (2018)  Quantitative research | Ireland | Multi-professions | 385 | Originally developed | Disasters in general | Multiple (e.g., planning, preparedness and response) | The Major Emergency Preparedness in Ireland Survey (MEPie) | - 53 items - Demographics - Baseline knowledge of major emergency planning - Knowledge of major emergency operations - Knowledge of core major emergency clinical response activities - Self-assessed clinical competence for a major emergency response - Perception of communication and coordination across principal response agencies | The Framework for Major Emergency Management | 2 | N | N | N | N | *** |
| Naser W and Saleem H (42) (2018)  Quantitative research | Yemen | Multi-professions | 531 | Originally developed | Disasters in general | Multiple (e.g., prevention, mitigation, response, recovery) | No name | - Knowledge (11 items) - Attitude (6 items) - Training and practice (3 items) - Anticipated disasters (2 items) - Demographic data (6 items) | No | 1 | 1 | N | N | N | **** |
| Good, L (20) (2009)  Mixed-methods | USA | Multi-professions | 452 | Originally developed | Specific disaster type (biologic emergencies) | Multiple (e.g., preparedness and response) | The Provider Response to Emergency Pandemic (PREP) | - Demographics (11 items)   Four Loss- subscales (loss of order, loss of safety, loss of trust, and loss of freedom) plus five exploratory items (31 items) | No | 2 | 1 | 1 | 1 | N | *********** |
| Marin S , et al. (43) (2020)  Quantitative research | Brazil | Nursing | 326 | Originally developed | Disasters in general | Specific (e.g., response) | Nurses’ Disaster Response Competencies Assessment Questionnaire (NDRCAQ) | - Care of the community (14 items) - Care of the individual and family (15 items) - Psychological support and care of vulnerable populations (12 items) | The Framework of Disaster Nursing Competencies (ICN) | 2 | N | 2 | N | N | ****** |
| Grimes D and Mendias E (44) (2009)  Quantitative research | USA | Nursing | 292 | Originally developed | Specific disaster type (bioterrorism and other infectious diseases) | Specific (e.g., response) | No name | - Personal/Professional Profile - Bioterrorism Knowledge (17 MCQ items) - Intention to Respond instrument (10 scenarios) | Icek Ajzen’s Theory of Planned Behavior | 2 | N | 2 | N | N | ****** |
| Rajesh G, et. al. (45) (2011)  Quantitative research | India | Dentistry | 125 | Originally developed | Disasters in general | Multiple (e.g., preparedness, response) | No name | - Knowledge (26 items) - Attitude (8 items) - Behavior (8 items) | No | 1 | N | 2 | N | N | ***** |
| Nofal A, et al. (46) (2021)  Quantitative research | KSA | Multi-professions | 1030 | Originally developed | Specific disaster type (disaster of a biological source) | Multiple (e.g., preparedness, response) | No name | - Demographics - Bioterrorism preparedness knowledge (50 items) - Perceived benefits and barriers to bioterrorism education (20 items) - Bioterrorism preparedness education receipt and responses (11 items) | No | 1 | N | 1 | N | N | **** |
| Alwidyan M, et. al. (47) (2020)  Quantitative research | Jordan | Emergency medical services (EMS)providers | 466 | Originally developed | Specific disaster type (Pandemic disasters) | Multiple (e.g., preparedness, response) | No name | - Demographics (8 items) - Attitude toward working during disease outbreaks (1 item) - Concerns for working during disease outbreaks (7 items) - Employer and the workplace (12 items) - Work obligation (8 items) - Role of family (2 items) | No | 1 | N | 1 | N | N | **** |
| Al-Hunaishi W , et. al. (48) (2019)  Quantitative research | Yemen | Multi-professions | 692 | Originally developed | Multiple, specified disasters (e.g., biological and natural disasters) | Specific (e.g., response) | No name | - Sociodemographic - professional and intrapersonal factors (including self-efficacy) - Willingness to participate in a disaster | Self-efficacy theory | 1 | N | 1 | N | N | **** |
| Ojukwu C, et. al. (49) (2021)  Quantitative research | Nigeria | Physiotherapists | 150 | Originally developed | Disasters in general | Multiple (e.g., prevention, mitigation, response, recovery) | No name | - Personal and occupational characteristics - knowledge and attitudes of specific roles of preparation and planning during the immediate response and recovery - Barriers to involvement in DM | No | 1 | N | 1 | N | N | **** |
| Randal D. Beaton and L. Clark Johnson (50) (2002)  Quantitative research | USA | Multi-professions | 455 (Baseline 209, Follow-up 246) | Originally developed | Multiple, specified disasters (Nuclear, Biological, or Chemical (NBC)/Weapons of Mass Destruction (WMD) | Specific (e.g., response) | Domestic Preparedness Questionnaire (DPQ) | - 32 items - Knowledge of (NBC)/ (WMD) awareness and operations content (27 items) - Level of hazardous materials training (1 item) - Total hours of prior NBC/WMD domestic terrorism preparedness training (1 item) - Level of "preparedness/competency" in responding to either a chemical, biological, or nuclear act of terrorism in the community (3 items) | No | 0 | N | 2 | 1 | N | ****** |
| Shwu-Ru Liou, et. al. (51) (2020)  Quantitative research | Taiwan | Nursing | 90 | Originally developed | Disasters in general | Not specified | DCCQ, ADSQ; not included), and MDEQ | - Demographic - DCCQ (26 items) - ADSQ (24 items) - MDEQ (3 items) | DCCQ: The ICN Framework of Disaster Nursing Competencies | DCCQ:  1  ADSQ:  0  MDEQ:  N | N | 2 | N | N | DCCQ:  *****  ADSQ:  ****  MDEQ:  *** |
| Hung M. et al. (52) (2021)  Quantitative research | China | Nursing | 157 | Originally developed | Disasters in general | Not specified | No name | - Disaster knowledge (15 items) - Willingness and perceived ability to respond to different catastrophic natural and human-caused disasters (6 items) - Concerns that influence willingness and perceived ability - Demographics | No | 1 | N | 1 | N | N | **** |
| Mosca N, et. al. (13) (2005)  Quantitative research | USA | Nursing | 125 | Originally developed | Specific disaster type (disaster of a biological source) | Multiple (e.g., prevention, mitigation, response, recovery) | No name | - 74 items - Knowledge of and training needs, role, in skills required for bioterrorism and emergency preparedness | - The CDC Bioterrorism and Emergency Preparedness Competencies for all Public Health Workers - The National Association of School Nurses’ Disaster Preparedness Guidelines for School Nurses | 1 | N | 0 | N | N | *** |
| Nofal, A et.al. (9) (2018)  Quantitative research | KSA | Multi-professions | 189 | Originally developed | Disasters in general | Multiple (e.g., preparedness, response) | No name | - Demographics - Knowledge about disaster management and preparedness (8 items) - Attitudes about disaster planning (11 items) - Current role and practices (3 items) - Familiarity towards emergence (EPIQ) | No | 1 | N | N | N | N | ** |
| Hayanga K, et al. (53) (2017)  Quantitative research | USA | Anesthesiologists | 270 | Originally developed | Multiple, specified disasters  (Natural disaster, pandemic influenza, and radiological dispersal device) | Multiple (e.g., prevention/mitigation, preparedness, response, recovery) | No name | - 95 items with 9 demographic items - Knowledge and skills, as well as attitudes and beliefs regarding the following: education and training, employee development, professional obligation, safety, psychological readiness, efficacy, personal preparedness, and willingness to respond | Extended Parallel Process Model (EPPM) | 1 | N | N | N | N | ** |
| Charney R, et. al. (54) (2015)  Quantitative research | USA | Multi-professions | 1822 | Originally developed | Specific disaster type (pandemic influenza and earthquake) | Multiple (e.g., preparedness and response) | No name | - 33 items with demographic items - Willingness to work - Ability to work - Perceived responsibility to work - Perceived job importance - Perceived barriers to working   Intent to work for scheduled and unscheduled shifts | No | 1 | N | N | N | N | ** |
| Ghahremani M, et al. (55) (2022)  Quantitative research | Iran | Nursing | 40 | Originally developed | Specific disaster type (disaster of a biological source) | Multiple (e.g., prevention, mitigation, response, recovery) | EPIQ (adopted) and bioterrorism performance measurement checklists (original) | - Demographics (8 items) - Bioterrorism knowledge scale (EPIQ; 44 items) - Six-station bioterrorism performance OSCE checklist | No | N | N | 1 | N | N | ** |
| Peñafrancia E. Ching & Rolando T. Lazaro (56) (2021)  Quantitative research | Philippine | Occupational therapy | 24 | Originally developed | Disasters in general | Multiple (e.g., prevention, mitigation, response, recovery) | No name | - Demographics and prior engagement in disaster-related work - Roles and responsibilities during (a) pre-disaster (preparedness; 7 items)   (b) immediate post-disaster (response; 10 items)  (c) rehabilitation and recovery phases (6 items)   - Degree of involvement in specific roles and responsibilities | No | 1 | N | N | N | N | ** |
| Berhanu N, et. al. (57) (2016)  Quantitative research | Southwest Ethiopia | Multi-professions | 377 | Originally developed | Multiple, specified disasters (drought, flood, epidemic diseases and fire and traffic accident) | Multiple (e.g., prevention, mitigation, response, recovery) | No name | - Socio-economic and demographic characteristics - Knowledge, experiences and training needs about disaster early warning, preparedness and response | - The CDC’s Competency - Hyogo Framework for Action 2005-2015 | 1 | N | N | N | N | ** |
| Jacobs-Wingo J, et. al. (58) (2019)  Mixed-methods | USA | Nursing | 7177 | Originally developed | Multiple, specified disasters (chemical, biological, radiological, nuclear, and explosive [CBRNE]) | Not specified | No name | - Number of items is not specified - Familiarity with emergence preparedness (EP) concepts and confidence in performing EP job duties | No | 0 | N | N | N | N | * |
| Kollek D, et. al. (59) (2009)  Quantitative research | Canada | Multi-professions | 1028 | Originally developed | Multiple, specified disasters  (Chemical, biological, radiological and nuclear [CBRN]) | Multiple (e.g., preparedness, response) | No name | - 28 items - Demographic - Theoretical and practical CBRN training | No | 0 | N | N | N | N | * |
| Hohman A. (60) (2008)  Quantitative research | USA | Nursing | 45 | Originally developed | Specific disaster type (Biological/chemical agents) | Multiple (e.g., prevention, mitigation, response, recovery) | No name | - Demographic (8 items) - Disaster Experience (3 items) - Education and Policy (15 items) - Future Considerations (4 items) | No | 0 | N | N | N | N | * |
| Stankovic C, et. al. (61) (2009)  Quantitative research | USA | Medicine | 590 | Originally developed | Specific disaster type (disaster of a biological source) | Multiple (e.g., prevention, preparedness, response) | No name | - Demographics - Bioterror preparedness (10 items) | No | 0 | N | N | N | N | * |
| Scott E, et. al. (62) (2008)  Quantitative research | USA | Dentistry | 370 | Originally developed | Specific disaster type (disaster of a biological source) | Multiple (e.g., prevention, mitigation, response, recovery) | No name | - 18 items - Dental professional’s opinions - Self-assessed knowledge bases - Actual knowledge bases - Need for training programs and CE courses | No | 0 | N | N | N | N | * |
| Suleiman M (63) (2022)  Quantitative research | Nigeria | Pharmacy | 102 | Originally developed | Disasters in general | Multiple (e.g., preparedness, response) | No name | - Demographics (5 items) - Knowledge of emergency preparedness and disaster management (15 items) | No | 1 | N | N | N | N | * |
| Schumacher L. et al (64) (2019)  Quantitative research | Europe-wide study | Pharmacy | 306 | Originally developed | Disasters in general | Multiple (e.g., prevention preparedness, response, recovery) | No name | - 55 items - Demographic - Prevention - Preparation - Response - Recovery - Drugs (information about emergency stockpile) - European risk perception | International Pharmaceutical Federation (FIP) guidelines for natural disasters | 0 | N | N | N | N | * |
| Reischl T. et al (12) (2008)  Quantitative research | USA | Environmental health professionals | 400 | Originally developed | Disasters in general | Multiple (e.g., prevention, mitigation, response, recovery) | The training needs assessment survey | - Demographics - Training topics needed - Level of training needed - Level of confidence for performing 20 emergency preparedness competencies - Three additional confidence for administrative competencies - Open-ended item about the survey topics | Columbia University School of Nursing competency set | 0 | N | N | N | N | * |

- MCQ: Multiple-Choice Questions
- NS: Not specified
- DM: Disaster Management
- DCCQ: Disaster Core Competencies Questionnaire
- ADSQ: Anticipatory Disaster Stress Questionnaire
- MDEQ: Motivation for Disaster Engagement Questionnaire
