# Supplementary material for: Instruments for the assessment of disaster management among healthcare professionals: a scoping review
Source: Front Public Health. 2025 Apr 11;13:1540743. doi: 10.3389/fpubh.2025.1540743 (PMC12021930; doi:10.3389/fpubh.2025.1540743)
Supplement: Supplementary file 4 [file Table_4.docx]

### Supplementary Material 4. Further details of the psychometric evaluation evidence of the included instruments

| **Author (Date)** | **Content** | **Response process** | **Internal structure** | **Relation to other variables** | **Consequence** | **Overall rating (*)** |
| --- | --- | --- | --- | --- | --- | --- |
| Wisniewski R, et. al. ^45^ (2004)  Quantitative research | - 1 - Qualitative research has informed instrument formation and previous literature - Assessment themes were listed with little or no reference to a theoretical basis | - N - 877 participants were involved in pilot testing of the instrument, but no information about the response rate - No info about the thought process or response errors | - 2 - Factor analysis (n=877) - Reduction of dimensions from 10 to 8 dimensions - The preparedness questions grouped as expected - The cumulative variance explained from the Equamax factor analysis was 73.5% - One measure of reliability: - Internal consistency: (n=877)   Cronbach’s alpha ranged from .827 to .94 | - N - No convergent or divergent correlation assessments conducted | - N - No description of consequences of assessment | ***** |
| ***Adapted by Garbutt S. et. al. ^73^ (2008)*** | - | - | - 2 - Factor analysis (n=776) - Dimensionality: PCA using a varimax rotation [One question with a factor loading of 0.4 was removed from consideration, some items were moved from the original dimension to the dimension where the items best fit, the cumulative variance explained was 73.5%] - One measure of reliability: - Internal consistency: (n=776)   Cronbach’s values ranging for the entire instrument= 0.97 (ranging from 0.83 to 0.94) | - 2 - Assessment of the overall predictability and the relative impact of each dimension was performed | - N - No description of consequences of assessment | ****** |
| Tichy et al ^4^ (2009)  Quantitative research | - 1 - Disaster preparedness education competencies for NPs identified in the literature - Assessment themes were listed with little or no reference to a theoretical basis - Reviewed and edited by panels of experts | - N - Response rate for the main study was provided: n=191/300 (64%) - No data regarding thought processes and analysis of responses - No discussion of response errors | - 1 - One measure of reliability: - Internal consistency:   Level of preparedness: a= 0.93  Level of preparedness for response: a= 0.93  Level of preparedness or disaster recovery: a= 0.91 | - N - No convergent or divergent correlation assessments conducted | - N - No description of consequences of assessment | **** |
| ***Adapted by Al Khalaileh M, et. al. ^74^ (2010)*** | - 2 - Beaton et al. (2000) Guidelines for the Process of Cross-Cultural Adaptation of Self-Report Measures was used - Three items were omitted - Translation of the original questionnaire into classical Arabic by two independent translators - The Arabic version was back translated into English by two independent translators and the back-translated version were compared with the original instrument by a committee of experts - Face validity was tested by an expert in the field, while the content validity was tested by a panel of experts | - 1 - Pilot study was conducted on 20 participants - The response rate for the actual use of the instrument: 474/600 (79%) - Minimal data regarding thought processes - No discussion of response errors | - 2 - Factor analysis: (n= 474/600 [79%]) - Three factors explained 64% of th variance: knowledge, skills and post disaster management - One measure of reliability: - Internal consistency (pilot test; n=20):   Knowledge: a=0.86  Skills: a=0.90  During and post disaster management: a=0.88  The overall reliability: a= 0.93   - Internal consistency (Main study) (n= 474/600 [79%]):   Knowledge: a=0.91  Skills: a=0.90  During and post disaster management: a=0.91  The overall reliability: a= 0.90 | - 1 - The correlations between the factors were performed and found satisfactory | - N - No description of consequences of assessment | ********** |
| ***Adapted by Suk Jung Han and Jiyoung Chun ^75^ (2010)*** | - 2 - The instrument development guidelines presented by DeVellis was followed - Translation and back-translation - Content Validity: - Same format as the original one - Seven academic and clinical experts conducted two rounds - Content validity index (CVI): Agreement of 80% or higher for all 46 items - Pilot study: - Format, linguistic expressions, and response time, a pilot survey was conducted among 55 nurses | - 1 - Pilot study was conducted on 55 participants - The response rate for the actual use of the instrument: 474/600 (79%) - Minimal data regarding thought processes - No discussion of response errors | - 2 - Factor analysis: - Exploratory factor analysis (EFA): n=248/497 (49.9%)   The instrument consisted of 34 items and five factors. The percentage of explained variance for each factor was 19.2% for recovery, 17.6% for bioterrorism and emergency response, 14.0% for disaster knowledge and information, 8.3% for disaster response, and 7.9% for disaster education and training, with a total explained variance of 66.949%   - Confirmatory factor analysis (CFA):   n=249/497 (50.1%)  The total number of items was reduced to 28 and the absolute fit indexes (2/df = 2.193, SRMR = 0.060, RMSEA = 0.069, GFI = 0.831, CFI = 0.927, and NFI = 0.875) aligned with the recommended values of GFI and CFI close to 1.00, SRMR 0.05, and RMSEA from 0.8 to 0.10   - One measure of reliability: - Internal consistency (preliminary survey):   The Cronbach’s alpha values were between 0.94 and 0.96   - Internal consistency (Actual survey): - The Cronbach’s alpha values of the 46 items was 0.96 | - 2 - Convergent Validity: - The composite reliability (CR) of the DPET-K items ranged from 0.659 to 0.937; and the Average Variance Extracted Estimate (AVE) ranged from 0.413 to 0.710 - Discriminant Validity: - All AVEs were observed to be greater than the square of the correlation coefficient between each factor - Concurrent Validity: - The correlations between the items of this instrument and Noh’s (2010) instrument ranged from r = 0.481(p< 0.001) to 0.740 (p< 0.001) | - N - No description of consequences of assessment | *********** |
| Al Thobaity A, et. al. ^46^ (2016)  Quantitative research | - 2 - A scoping review conducted - Assessment themes were listed with reference to explicit theoretical/conceptual basis (e.g., the ICN disaster-nursing framework) - A well-defined process for creating and reviewing items - Systematic item review (content and face validity) | - N - Response rate: - Test-retest reliability: 29/33 (87.87%) - PCA: 132/200 (66%) - No data regarding thought processes and analysis of responses - No discussion of response errors | - 2 - PCA of the 93 questionnaire items revealed 49 redundant items (which were deleted) and 3 factors with eigenvalues of >1. The remaining 44 items accounted for 77.3% of the total variance. - Measures of reliability: - Test-retest reliability: (n=29/33 [87.87%])   Satisfactory correlation between time 1 and time 2 of the total scale (r=0.76, p<0.001)   - Internal consistency: (n= 132/200 [66%])   Overall Cronbach’s alpha= 0.96, Factor 1=0.98, Factor 2= 0.92 and Factor 3= 0.86   - The variation in responses to specific items among subgroups is not reported | - N - Convergent and discrimination validity was not performed | - N - No description of consequences of assessment | ****** |
| Al-Ziftawi N, et. al. ^25^ (2020)  Quantitative research | - 1 - Assessment themes were listed with little reference to a theoretical basis (was developed from a review of the literature of previous studies), and a poorly defined process for creating and reviewing items | - N - Five participants were involved in pilot testing of the instrument, but no information about the response rate - Feedback was provided by participants, but no info about the thought process or response errors | - 1 - No factor analysis conducted - One measure of reliability: - Internal consistency: (n=5). Cronbach’s alpha for the overall instrument= 0.909, knowledge=0.627, attitude=0.897, and readiness to practice=0.734 | - N - No convergent or divergent correlation assessments conducted | - N - No description of consequences of assessment | **** |
| Veenema T, et al ^47^ (2018)  Quantitative research | - 2 - The concept of major emergency was based on the Framework for Major Emergency Management - Assessment themes were listed - Conceptual domains were identified through a review of the existing peer-reviewed literature, previously published questionnaires - Conceptual domains were refined through focus groups with emergency HCPs - Pretested and edited by 7 subject matter experts - Pilot-tested with HCPs with a resultant content validity index of 0.92 | - N - Response rate: - For the instrument development and pilot testing: Not mentioned - For the main study: n=385 (59% for hospital-based providers, 10% for fire-based emergency medical service, and unknown for the ambulance service ) - No data regarding thought processes and analysis of responses - No discussion of response errors | - N - No factor analysis or reliability assessments were conducted | - N - No convergent or divergent correlation assessments conducted | - N - No description of consequences of assessment | *** |
| Naser W and Saleem H ^48^ (2018)  Quantitative research | - 1 - Instrument development was based on relevant literature of similar studies - Assessment themes were listed with little or no reference to a theoretical basis | - 1 - Pilot study was conducted on 50 participants - The instrument was completed by 531 in the main study - Minimal data regarding thought processes - No discussion of response errors | - N - No factor analysis or reliability analysis conducted | - N - No convergent or divergent correlation assessments conducted | - N - No description of consequences of assessment | **** |
| Good, L ^30^ (2009)  Mixed-methods | - 2 - The PREP Instrument was constructed by an expert panel based on literature review - Assessment themes were listed with reference to explicit theoretical/conceptual basis (conceptual model was drawn from the four loss domains) - A well-defined process for creating and reviewing items - Systematic item review (content and face validity) | - 1 - The PREP Instrument was pretested with a focus group and feedback was described and used to review the instrument - Minimal data regarding the thought process | - 2 - Factor analysis: - Exploratory factor analysis (EFA):   Factor extraction and rotation allowed items to be loaded onto 6 factors   - One measure of reliability: - Internal consistency (n=452):   The Cronbach’s alpha values for 20 items out of 31 within the five original Loss-subscale was between 0.81 and 0.85 | - 2 - Correlations assessments were performed to distinguish/predict RTW | - N - No description of consequences of assessment | *********** |
| Marin S , et al. ^49^ (2020)  Quantitative research | - 2 - Competencies were extracted from the Framework of Disaster Nursing Competencies published by the International Council of Nurses - Content validity: 8/20 (40%) - Content validity index (CVI): 0.88 (SD= 0.12) - Intraclass correlation coefficient (ICC): 0.92 (SD=0.04) | - N - Response rate for different assessments were provided - No data regarding thought processes and analysis of responses - No discussion of response errors | - 2 - Factor analysis: (n= 326/608 [53%]) - 3 domains with eigenvalues: 16.7, 5.5, and 2.3, respectively - Variance % explained by the 3 factors: 19.6%, 36.9%, and 53.1%, respectively (Kaiser-Meyer-Olkin test value 0.95; Bartlett’s test x2= 10,123; P < 0.001) - Measures of reliability: - Test-retest reliability: (n= 21/32 [65%])   No significant difference between the test and retest scores  ICC > 0.7   - Internal consistency: (n= 326/608 [53%])   Overall Cronbach’s alpha= 0.96  Cronbach’s alpha for the 3 factors >0.92 | - 1 - Construct validity associations were evaluated | - N - No description of consequences of assessment | ******** |
| Grimes D and Mendias E ^50^ (2009)  Quantitative research | - 2 - Assessment themes were listed with reference to a theoretical basis - Content was revised by faculty members | - N - The instrument was pilot tested - The response rate for the actual use of the instrument: 292/313 (93.2%) - No data regarding thought processes and analysis of responses - No discussion of response errors | - 2 - Two measures of reliability: - The inter-item reliability (KR20) on the 17-item Knowledge of Bioterrorism instrument was 0.79 on the first 100 participants in the study - The inter-item reliability(Cronbach’s a) for all participants on the 10 scenarios comprising the intentions to respondwas 0.913 | - N - No convergent or divergent correlation assessments conducted | - N - No description of consequences of assessment | ****** |
| Rajesh G, et. al. ^51^ (2011)  Quantitative research | - 1 - The instrument was developed from four sources: theory, research, observation, and expert opinion - Assessment themes were listed with little or no reference to a theoretical basis | - N - The instrument was pilot tested on 35 - The response rate for the actual use of the instrument: 125/135 (92.5%) - No data regarding thought processes and analysis of responses - No discussion of response errors | - 2 - Two measures of reliability: - The internal reliability:   Cronbach’s for knowledge, attitude, and behavior were 0.72 and 0.86, 0.86   - Split-half reliability:   Values for knowledge, attitude, and behavior were 0.82 and 0.87 | - N - No convergent or divergent correlation assessments conducted | - N - No description of consequences of assessment | ***** |
| Nofal A, et al. ^52^ (2021)  Quantitative research | - 1 - Instrument was developed based on a review of the literature of related studies - Assessment themes were listed with little or no reference to a theoretical basis | - N - The instrument was pilot tested on 30 participants - No data regarding thought processes and analysis of responses - No discussion of response errors | - 1 - One measure of reliability: - Internal consistency reliability:   Cronbach’s alpha was 0.86 for the perceived benefits, 0.91 for the perceived barriers, and 0.88 for bioterrorism knowledge | - N - No convergent or divergent correlation assessments conducted | - N - No description of consequences of assessment | **** |
| Alwidyan M, et. al. ^53^ (2020)  Quantitative research | - 1 - An expert panel developed a questionnaire - Assessment themes were listed with little or no reference to a theoretical basis | - N - The instrument was pilot tested on 10 participants - The response rate for the actual use of the instrument: 466/500 (93.2%) - No data regarding thought processes and analysis of responses - No discussion of response errors | - 1 - Factor analyses and internal reliability analyses were performed whenever appropriate to reduce variables and validate categories - Findings of factor analysis is not described - One measure of reliability: - Internal consistency reliability:   Cronbach’s alpha for ‘concerns for working during disease outbreaks’ was 0.834  Cronbach’s alpha for ‘employer and the workplace’ was 0.826  Cronbach’s alpha for ‘work obligation’ was 0.890 | - N - No convergent or divergent correlation assessments conducted | - N - No description of consequences of assessment | **** |
| Al-Hunaishi W , et. al. ^54^ (2019)  Quantitative research | - 1 - The instrument was developed based on information from previous studies - Content validity: - By national and international experts - Some items were deleted and other were reworded. - The revised instrument was then forward and backward translated from English to Arabic and from Arabic to English - Face validity: - Three HCWs were asked to give their opinion | - N - The instrument was pilot tested on 20 participants - The response rate for the actual use of the instrument: 767/1093 (70.2%) - No data regarding thought processes and analysis of responses - No discussion of response errors | - 1 - One measure of reliability: - Internal consistency reliability:   Cronbach’s alpha for self- efficacy was 0.801 | - N - No convergent or divergent correlation assessments conducted | - N - No description of consequences of assessment | **** |
| Ojukwu C, et. al. ^55^ (2021)  Quantitative research | - 1 - Assessment themes are listed, with little reference to a theoretical basis - Content validity and face validity by four experts with no data discussed | - N - The instrument was pilot tested on 10 participants - The response rate for the actual use of the instrument: 157/180 (87.2%) - No data regarding thought processes and analysis of responses - No discussion of response errors | - 1 - One measure of reliability: - Test-retest reliability: - Intraclass correlation analysis r=0.896 (p=0.001) | - N - No convergent or divergent correlation assessments conducted | - N - No description of consequences of assessment | **** |
| Randal D. Beaton and L. Clark Johnson ^56^ (2002)  Quantitative research | - 0 - Instrument developed based in the training material - Assessment themes are listed | - N - The instrument was pilot tested on 78 participants - Psychometric properties are tested on 206 baseline and 2046 follow-up samples - No data regarding thought processes and analysis of responses - No discussion of response errors | - 2 - Two measures of reliability: - Internal consistency reliability:   Cronbach a for the total DPQ score= 0.89 [baseline sample (n = 206)]   - Test-retest reliability:   DPQ total score= 0.49 for the Not-DP-Trained sample  (n= 75) and 0.64 for the DP-Trained sample (n = 80).  The six-month test-retest reliabilities for the Not-DP-Trained sample for the self-appraised competencies to respond to biological, chemical, and nuclear events (in their communities)= 0.57,0.54, and 0.45, respectively.   - Parallel test-retest reliabilities for these competency items in the DP-Trained sample were 0.48,0.63, and 0.62, respectively | - 1 - Correlations assessment between the perceived competency item and questionnaire section were performed and were satisfactory | - N - No description of consequences of assessment | ****** |
| Shwu-Ru Liou, et. al. ^57^ (2020)  Quantitative research | DCCQ:   - 1 - The instrument was developed based on the ICN framework - Assessment themes were listed with reference to a theoretical basis - No information regarding reviewing and refining the instrument   ADSQ:   - 0 - The instrument was developed based on literature - Assessment themes were not listed - No information regarding reviewing and refining the instrument   MDEQ:   - N - No information about the source of instrument development - Assessment themes were not listed - No information regarding reviewing and refining the instrument | - N for all instruments - No information about pilot testing - The response rate for the actual use of the instrument: (86.54) - No data regarding thought processes and analysis of responses - No discussion of response errors | DCCQ:   - 2 - Factor analysis:   Principal component analysis for the validity showed that 62.50% of the variance of disaster nursing competence could be explained by the DCCQ   - One measure of reliability: - Internal consistency:   The Cronbach’s alpha was 0.95  ADSQ:   - 2 - Factor analysis:   Principal component analysis showed that 44.87% of the variance of the anticipatory disaster stress could be explained by the ADSQ   - One measure of reliability: - Internal consistency:   The Cronbach’s alpha was 0.74  MDEQ:   - 2 - Factor analysis:   Principal component analysis showed that 67.09% of the variance of the motivation in disaster engagement could be explained by the MDEQ   - One measure of reliability: - Internal consistency:   Cronbach’s alpha was 0.75 | - N for all instruments - No convergent or divergent correlation assessments conducted | - N for all instruments - No description of consequences of assessment | DCCQ:  *****  ADSQ:  ****  MDEQ:  *** |
| Hung M. et al. ^58^ (2021)  Quantitative research | - 1 - Assessment themes are listed, with little reference to a theoretical basis - Content validity: - Four experienced nurses - The average scale content validity index (SCVI) was 0.96 | - N - The instrument was pilot tested on 20 participants - The response rate for the actual use of the instrument: 157/180 (87.2%) - No data regarding thought processes and analysis of responses   No discussion of response errors | - 1 - One measure of reliability: - Internal consistency reliability:   Cronbach’s alpha for ‘perceived ability’ pre- and post-course= 0.89 and ‘willingness’ pre- and post-course= 0.82. | - N - No convergent or divergent correlation assessments conducted | - N - No description of consequences of assessment | ***** |
| Mosca N, et. al. ^59^ (2005)  Quantitative research | - 1 - Instrument development was based on competencies derived from publications by the Centers for Disease Control and Prevention and National Association of School Nurses   Assessment themes were listed with no reference to a theoretical basis | - N - The instrument was pilot tested with no discussion - The response rate for the actual use of the instrument: (80/125 [64%]) - No data regarding thought processes and analysis of responses - No discussion of response errors | - 0 - One measure of reliability: - Internal consistency reliability - Cronbach’s alpha were 0.60 or higher (unacceptable) | - N - No convergent or divergent correlation assessments conducted | - N - No description of consequences of assessment | *** |
| Nofal, A et.al. ^19^ (2018)  Quantitative research | - 1 - Instrument was developed based on a review of the literature of related studies - Assessment themes are listed, with no reference to a theoretical basis, poorly defined process for creating these items | - N - The response rate for the actual use of the instrument: 189/250 (75.6%) - No data regarding thought processes and analysis of responses - No discussion of response errors | - N - No factor analysis or reliability analysis conducted | - N - No convergent or divergent correlation assessments conducted | - N - No description of consequences of assessment | ** |
| Hayanga K, et al. ^60^ (2017)  Quantitative research | - 1 - The instrument was developed based on previous literature and EPPM model - Assessment themes were listed with reference to a theoretical basis   No information about instrument revision | - N - No information about pilot testing - The response rate for the actual use of the instrument: (47% and 51% response rate) - No data regarding thought processes and analysis of responses   No discussion of response errors | - N - No factor analysis or reliability analysis conducted | - N - No convergent or divergent correlation assessments conducted | - N - No description of consequences of assessment | ** |
| Charney R, et. al. ^61^ (2015)  Quantitative research | - 1 - Instrument development was based on relevant literature of similar studies - Assessment themes were listed with no reference to a theoretical basis - Content validity: - CVI: No items had a CVI <0.80, but no data discussed | - N - The instrument was pilot tested on 10 participants with no discussion - No data regarding thought processes and analysis of responses - No discussion of response errors | - N - No factor analysis or reliability analysis conducted | - N - No convergent or divergent correlation assessments conducted | - N - No description of consequences of assessment | ** |
| Ghahremani M, et al. ^62^ (2022)  Quantitative research | - N - The source of instrument development (OSCE checklist) is not mentioned - Assessment themes were not clearly listed - Content validity: It was approved by 10 faculty members and experts, but no discussion | - N - No data regarding thought processes and analysis of responses - No discussion of response errors | - 1 - One measure of interrater reliability:   Pearson’s correlation coefficient was r=0.97 | - N - No convergent or divergent correlation assessments conducted | - N - No description of consequences of assessment | ** |
| Peñafrancia E. Ching & Rolando T. Lazaro ^63^ (2021)  Quantitative research | - 1 - Assessment themes are listed, with little reference to a theoretical basis - Item review (content and face validity), but no data presented | - N - The instrument was pilot tested on 15 participants - The response rate for the actual use of the instrument: 24/31 (77.4%) - No data regarding thought processes and analysis of responses - No discussion of response errors | - N - No factor analysis or reliability analysis conducted | - N - No convergent or divergent correlation assessments conducted | - N - No description of consequences of assessment | ** |
| Berhanu N, et. al. ^64^ (2016)  Quantitative research | - 1 - Instrument development was based on relevant literature of similar studies - Assessment themes were listed with little reference to a theoretical basis | - N - The instrument was pilot tested with no discussion - The response rate for the actual use of the instrument: (93%) - No data regarding thought processes and analysis of responses   No discussion of response errors | - N - No factor analysis or reliability analysis conducted | - N - No convergent or divergent correlation assessments conducted | - N - No description of consequences of assessment | ** |
| Jacobs-Wingo J, et. al. ^65^ (2019)  Mixed-methods | - 0 - The instrument was designed based on focus group findings - Assessment themes are listed   Content validity with no data | - N - The instrument was pilot tested with no discussion - The response rate for the actual use of the instrument: (36%) - No data regarding thought processes and analysis of responses   No discussion of response errors | - N - No factor analysis or reliability analysis conducted | - N - No convergent or divergent correlation assessments conducted | - N - No description of consequences of assessment | * |
| Kollek D, et. al. ^66^ (2009)  Quantitative research | - 0 - The instrument was designed based on literature review - Assessment themes are listed   Content review with no discussion | - N - No data about the response rate - No data regarding thought processes and analysis of responses - No discussion of response errors | - N - No factor analysis or reliability analysis conducted | - N   No convergent or divergent correlation assessments conducted | - N - No description of consequences of assessment | * |
| Hohman A. ^67^ (2008)  Quantitative research | - 0 - The source of instrument development is not mentioned - Content validity and face validity with no data discussed | - N - The instrument was pilot tested on 5 participants - The response rate for the actual use of the instrument: 45/241 (18.6%) - No data regarding thought processes and analysis of responses - No discussion of response errors | - N - No factor analysis or reliability analysis conducted | - N - No convergent or divergent correlation assessments conducted | - N - No description of consequences of assessment | * |
| Stankovic C, et. al. ^68^ (2009)  Quantitative research | - 0 - The instrument was designed based on AAP policy statement - Content validity - Listing items only | - N - The instrument was pilot tested on 25 participants - The response rate for the actual use of the instrument: 590/1000 (59%) - No data regarding thought processes and analysis of responses - No discussion of response errors | - N - No factor analysis or reliability analysis conducted | - N - No convergent or divergent correlation assessments conducted | - N - No description of consequences of assessment | * |
| Scott E, et. al. ^69^ (2008)  Quantitative research | - 0 - The instrument was designed based on previous literature - Assessment themes are listed - Face validity with no data | - N - The instrument was pilot tested on 15 participants with no discussion - No data about the response rate - No data regarding thought processes and analysis of responses - No discussion of response errors | - N - No factor analysis or reliability analysis conducted | - N - No convergent or divergent correlation assessments conducted | - N - No description of consequences of assessment | * |
| Suleiman M ^70^ (2022)  Quantitative research | - 0 - The instrument was developed based on previous studies - Assessment themes are listed - Content and face validity but no discussion | - N - No data about the response rate - No data regarding thought processes and analysis of responses - No discussion of response errors | - N - No factor analysis or reliability analysis conducted | - N - No convergent or divergent correlation assessments conducted | - N - No description of consequences of assessment | * |
| Schumacher L. et al ^71^ (2019)  Quantitative research | - 0 - The instrument was developed based on FIP recommendations - Assessment themes are listed | - N - The response rate for the actual use of the instrument: 306/339 (90.3%) - No data regarding thought processes and analysis of responses - No discussion of response errors | - N - No factor analysis or reliability analysis conducted | - N - No convergent or divergent correlation assessments conducted | - N - No description of consequences of assessment | * |
| Reischl T. et al ^22^ (2008)  Quantitative research | - 0 - The instrument was designed based on competencies set and expert discussion - Assessment themes are listed | - N - The instrument was pilot tested with no discussion - No data regarding thought processes and analysis of responses - No discussion of response errors | - N - No factor analysis or reliability analysis conducted | - N - No convergent or divergent correlation assessments conducted | - N - No description of consequences of assessment | * |
